# Utilizing milk from pooling facilities as a novel approach for foot‐and‐mouth disease surveillance

**DOI:** 10.1111/tbed.13487

**Published:** 2020-02-04

**Authors:** Bryony Armson, Antonello Di Nardo, Dickson M. Nyaguthii, Beatriz Sanz‐Bernardo, Philip M. Kitala, Eunice Chepkwony, Valerie Mioulet, Donald P. King, Nicholas A. Lyons

**Affiliations:** ^1^ The Pirbright Institute Surrey UK; ^2^ Boyd Orr Centre for Population and Ecosystem Health Institute of Biodiversity, Animal Health and Comparative Medicine College of Medical, Veterinary and Life Sciences University of Glasgow Glasgow UK; ^3^ Department of Public Health, Pharmacology and Toxicology Faculty of Veterinary Medicine University of Nairobi Nairobi Kenya; ^4^ Foot‐and‐Mouth Disease Laboratory State Department of Livestock Embakasi Nairobi Kenya; ^5^ European Commission for the Control of Foot‐and‐Mouth Disease (EuFMD) Animal Production and Health Division Food and Agriculture Organization of the United Nations Rome Italy

**Keywords:** endemic systems, foot‐and‐mouth disease, Kenya, pooled milk, rRT‐PCR, surveillance

## Abstract

This study investigated the potential of pooled milk as an alternative sample type for foot‐and‐mouth disease (FMD) surveillance. Real‐time RT‐PCR (rRT‐PCR) results of pooled milk samples collected weekly from five pooling facilities in Nakuru County, Kenya, were compared with half‐month reports of household‐level incidence of FMD. These periodic cross‐sectional surveys of smallholder farmers were powered to detect a threshold household‐level FMD incidence of 2.5% and collected information on trends in milk production and sales. FMD virus (FMDV) RNA was detected in 9/219 milk samples, and using a type‐specific rRT‐PCR, serotype SAT 1 was identified in 3/9 of these positive samples, concurrent with confirmed outbreaks in the study area. Four milk samples were FMDV RNA‐positive during the half‐months when at least one farmer reported FMD; that is, the household‐level clinical incidence was above a threshold of 2.5%. Additionally, some milk samples were FMDV RNA‐positive when there were no reports of FMD by farmers. These results indicate that the pooled milk surveillance system can detect FMD household‐level incidence at a 2.5% threshold when up to 26% of farmers contributed milk to pooling facilities, but perhaps even at lower levels of infection (i.e., below 2.5%), or when conventional disease reporting systems fail. Further studies are required to establish a more precise correlation with estimates of household‐level clinical incidence, to fully evaluate the reliability of this approach. However, this pilot study highlights the potential use of this non‐invasive, routinely collected, cost‐effective surveillance tool, to address some of the existing limitations of traditional surveillance methods.

## 1 INTRODUCTION

In regions where foot‐and‐mouth disease (FMD) is endemic such as East Africa, surveillance is often dependent upon the recognition and reporting of clinical cases by farmers and livestock workers (Bates et al., 2003; Machira & Kitala, 2017; Picado et al., 2011). These passive surveillance activities may be supplemented by targeted case finding or serological surveys, but are generally infrequent due to the costs and labour involved (Hadorn & Stark, 2008; Kasanga et al., 2012). Additionally, the capacity for undertaking outbreak investigation and collection of clinical specimens in resource‐limited countries is often restricted (Kasanga et al., 2012). Furthermore, farmers may be deterred from informing veterinary services of suspected disease due to the repercussions of imposed control measures. As a consequence, FMD is often under‐reported, and it is often difficult to determine the true incidence of the disease, particularly when notification relies only on passive surveillance (Knight‐Jones, McLaws, & Rushton, 2016; Vosloo, Bastos, Sangare, Hargreaves, & Thomson, 2002).

Serum samples can be used to measure FMD virus (FMDV)‐specific antibodies to assess the prevalence of FMD. However, collection of sera is invasive, and results are retrospective and less reliable for characterizing serotypes (or even viral lineages) circulating in a region (Hedger, Barnett, Gradwell, & Travassos Dias, 1982; Morris et al., 2018) and are often difficult to interpret in older animals. Typically, vesicular epithelium and fluid samples are collected so that detection and characterization of the causal FMDV lineage may be carried out to inform the epidemiological situation and control strategies (Paton, Sumption, & Charleston, 2009). However, these sample types may only be collected from acutely infected animals and are invasive and labour‐intensive to collect. Moreover, animals with sub‐clinical FMD infection are unlikely to be identified, but may still play an important role in disease transmission (Sutmoller & Casas, 2002).

Milk represents a routinely collected, non‐invasive alternative to the traditional diagnostic sample types for FMDV. Previous studies have demonstrated that the mammary gland is highly susceptible to FMDV replication (Burrows, Mann, Greig, Chapman, & Goodridg, 1971) and that FMDV can be detected by real‐time reverse transcription polymerase chain reaction (rRT‐PCR) assays in milk from experimentally infected cattle, before and during the appearance of clinical signs, and up to 28 days post‐infection (Armson et al., 2018; Reid et al., 2006). FMDV RNA has also been detected in milk samples from naturally infected cattle (Armson et al., 2019; Ranjan et al., 2016; Saeed et al., 2011) and buffaloes (Ahmed et al., 2017) in endemic settings. Furthermore, using individual milk samples collected from naturally infected cattle in northern Tanzania, it has been demonstrated that VP1 sequences could be obtained, corresponding with those generated from clinical samples collected from lesions from the same animal (Armson et al., 2019). Concordant results were also generated between typing data from these milk samples and confirmed reports from outbreak investigations, highlighting the potential for using milk sampling from individual cattle as an alternative sampling approach.

Pooled milk has been used for the surveillance of a number of other diseases, including bovine viral diarrhoea (Drew, Yapp, & Paton, 1999; Dubovi, 2010; Hill, Reichel, & Tisdall, 2010), brucellosis (Chand, Rajpurohit, Malhotra, & Poonia, 2005; Hamdy & Amin, 2002) and Q fever (Bauer et al., 2015; Kim, Kim, Lafferty, & Dubovi, 2005), allowing cost‐effective disease surveillance at the herd level. The potential for applying this approach for the detection and surveillance of FMDV has been identified in previous studies. For example, detection of FMDV RNA has been demonstrated at a dilution of up to 10^−7^, and assuming a negligible reduction in milk yield, it could theoretically be possible to identify one acutely infected milking cow in a herd of up to 1,000 using pooled milk sampling (Armson et al., 2018). Additionally, simulation modelling (Garner et al., 2016; Kompas et al., 2017; Thurmond & Perez, 2006) suggested pooled milk could be a useful tool in enhancing a surveillance system for FMD and considered for regional FMD surveillance.

Foot‐and‐mouth disease has been described as a high impact disease among pastoralists in Kenya (Nthiwa, Alonso, Odongo, Kenya, & Bett, 2019; Onono, Wieland, & Rushton, 2013), and several studies have examined the impact of FMD outbreaks on large scale farms (Kimani, Mwirigi, & Murithi, 2005; Lyons, Alexander, et al., 2015; Lyons, Stärk, et al., 2015; Mulei, Wabacha, & Mbithi, 2001). There is a requirement for improved disease surveillance particularly on smallholder farms (Knight‐Jones et al., 2016). In Kenya, smallholder dairy farmers contribute an estimated 70%–80% of all milk sold to the dairy production chain, which, directly or indirectly, supplies consumers milk pooling facilities or private processors (Karanja, 2003; Omore, Muriuki, Kenyanjui, Owango, & Staal, 1999; Rademaker, Koech, Jansen, & Lee, 2016). It is possible that milk from these smallholder farmers may represent a useful resource for FMD surveillance. This pilot study aimed (a) to validate the use of pooled milk as a sample matrix for FMDV detection and characterization and (b) to assess the usefulness of pooled milk as a cost‐effective, non‐invasive alternative for FMD surveillance in Kenya whilst improving knowledge on milk production and selling trends. Finally, results obtained by FMDV rRT‐PCR of milk samples from pooling facilities were tested for correlation with reports of clinical disease from surveys of smallholder farmers.

## 2 MATERIALS AND METHODS

### 2.1 Study area and population

**Figure 1:**
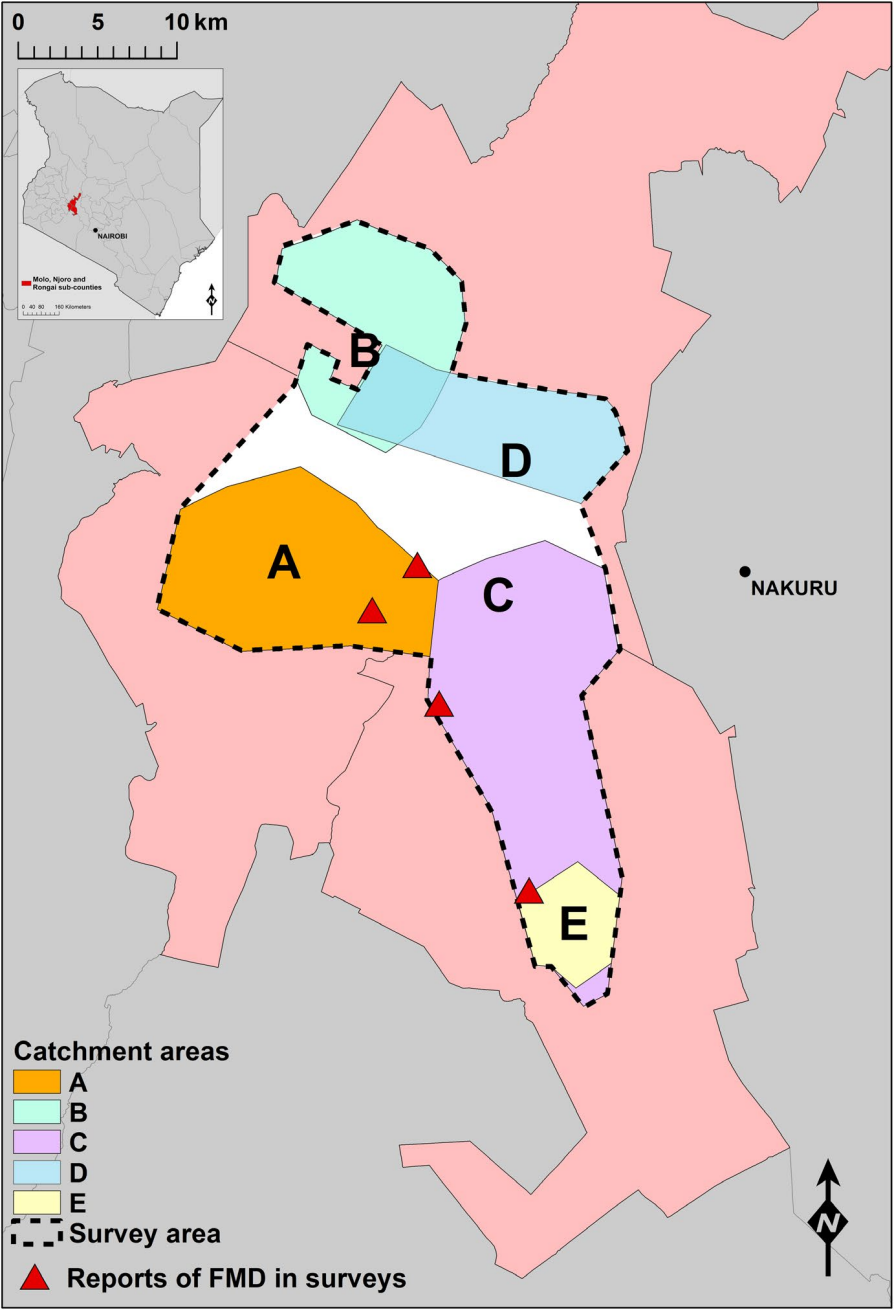
Map of the study area located in Molo, Rongai and Njoro sub‐counties of Nakuru County, Kenya. Capitalized letters indicate the location of each milk pooling facility (A–E). Catchment areas for each facility are colour‐coded. Catchment area D overlaps that of B. Additionally, catchment area E overlays C. The survey area is bordered with a dashed black line, and the white area indicates parts of the study area that were not within the catchment areas of any of the milk pooling facilities. Red triangles indicate the locations of smallholder farmers where FMD was reported during Survey 2. FMD, foot‐and‐mouth disease [Colour figure can be viewed at wileyonlinelibrary.com]

The study area and population have been described previously (Nyaguthii et al., 2019). Briefly, the study area consisted of neighbouring catchment areas of five milk pooling facilities that were recruited for sample collection, located within Molo, Njoro and Rongai sub‐counties of Nakuru County, Kenya (Figure 1). This area was selected due to the large numbers of FMD susceptible livestock present, regular outbreaks of FMD and the presence of a large number of smallholder dairy farmers. The milk pooling facilities were approached, and informed consent was provided prior to participation in the study. Catchment areas were constructed with the guidance of facility managers using Google Earth (Google Inc., USA), and as some of the catchment areas bordered or overlapped each other, a single spatial polygon layer was created using QGIS version 2.18.10 (QGIS Development Team) to define the entire study area (Figure 1). Sample collection was organized so that one person could visit all facilities within a few hours.

### 2.2 Study design

The primary goal of the study was the detection of viral RNA by rRT‐PCR in samples collected from milk pooling facilities to be compared with the presence of clinical FMD above a defined threshold in the entire study area. In order to achieve this objective, two concurrent studies were performed:

1. Repeat cross‐sectional surveys of smallholder dairy farmers to determine FMD incidence in the entire study area and improve knowledge on milk production and selling trends in the catchment areas of the five milk pooling facilities.
2. The collection of pooled milk samples from the five milk pooling facilities, at 45 weekly time points for FMDV detection by rRT‐PCR.

#### 2.2.1 Cross‐sectional surveys for clinical disease

A baseline survey (referred to as S1) conducted between 16 November and 1 December 2016, with the main aim of improving knowledge on the FMD susceptible population has been described previously (Nyaguthii et al., 2019). In the present study, S1 provided information on herd size, milk production and milk sales only, as results on FMD clinical incidence from this survey have already been described. The sample size for S1 was based on an expected proportion of households being affected in the previous six months of 15%.

Two subsequent surveys were carried out during 23–29 March 2017 (referred to as S2) and 20 to 26 September 2017 (referred to as S3). Due to a limitation on resources, S2 and S3 surveys were powered to detect a threshold household‐level FMD incidence of 2.5% based on perfect test sensitivity and specificity, a 95% confidence interval and an infinite study population. Based on these parameters, an online epidemiological calculator EpiTools (http://epitools.ausvet.com.au) (Sergeant, 2019) was used to define the number of households required for each survey as 120. The questionnaire used for S2 and S3 is available in Appendix S1.

The eligibility criteria for the study population were smallholder dairy farmers that owned at least one, but no more than 50 cattle, and had cattle located within the premises. A systematic set of spatial points was randomly generated within the study area polygon using QGIS version 2.18.10 (QGIS Development Team). During the field surveys, the closest smallholder dairy farm to a randomly generated coordinate that fitted the eligibility criteria was surveyed. This was considered the optimal approach in the absence of a sampling frame or recent census data (with the last being done in 2009). The limitation of this approach is the assumption that smallholder farmers were evenly distributed throughout the study area. Questionnaire data were collected using the EpiCollect+ mobile phone application (Aanensen, Huntley, Feil, Al‐Own, & Spratt, 2009).

To determine FMD clinical incidence for S2 and S3, farmers were asked whether they had observed FMD on their farm sequentially since the last survey. Farmers were asked to provide an indication of the time of the outbreak (either the first half or second half of the month) to allow FMD household‐level clinical incidence to be estimated in two week blocks of time (see statistical analysis section for further details). The case definition for FMD was defined as farmers observing at least two of the clinical signs listed by the African Union—Inter African Bureau for Animal Resources (AU‐IBAR, 2014) in at least one of their animals.

#### 2.2.2 Pooled milk samples

Milk samples were collected for 45 weeks (16/11/2016–20/09/2017) from each of the five recruited milk pooling facilities (denoted A–E), once per week. Milk was collected in 15‐mL sterile falcon tubes directly from the 5,000‐L capacity pooling tank for facilities A and B. A pooling tank was either not available or in use for facilities C, D and E, instead with milk being pooled into 50‐L cans (<25 cans per facility). Therefore, to obtain a representative milk sample at these facilities, 3 ml of milk was taken from each can, pooled in a jug and mixed, and a 15 ml aliquot was taken. At each collection, the total volume of milk in the tank/cans and the number of farmers contributing were recorded.

Immediately upon collection, all milk samples were stored on ice for a maximum of 4 hr during transportation to a local −20°C storage facility that was equipped with a temperature monitor. Milk samples were stored for up to nine months (depending on sample collection date) and then shipped on dry ice to The Pirbright Institute (TPI) for subsequent laboratory analysis.

### 2.3 Laboratory testing of pooled milk samples

RNA extraction and the pan‐serotypic rRT‐PCR assay were carried out in duplicate on all milk samples using an optimized method as previously described (Armson et al., 2018). Briefly, RNA was extracted from whole milk samples using the MagMAX™ Pathogen RNA/DNA Kit (Applied Biosystems^®^) on a MagMAX™ Express 96 Extraction Robot (Applied Biosystems^®^). rRT‐PCR assays were performed using the reagents, parameters and thermal cycling conditions previously reported (Shaw et al., 2007), with primers and probes targeting the conserved 3D region of the FMDV genome (Callahan et al., 2002). Any milk sample with a *C*_T_ value of ≤ 50 was considered positive and was subsequently tested by the East Africa (EA) typing rRT‐PCR assays [O, A, Southern African Territories (SAT) 1 and SAT 2], as described previously (Bachanek‐Bankowska et al., 2016). However, the fluorophores used on each probe were modified to A—Cy5, SAT 1—JOE^TM^, SAT 2—TAMRA^TM^. This modification does not affect the sensitivity of the rRT‐PCR assays (data not shown). Positive samples for the EA rRT‐PCR typing assays were also defined as those with a *C*_T_ value of ≤50.

FMD virus cell culture isolates were obtained from archival stocks held in the FAO/OIE World Reference Laboratory for foot‐and‐mouth disease repository (WRLFMD), TPI, UK. Cell culture isolate O/SAU/1/2016 was used to prepare positive control material for the pan‐serotypic rRT‐PCR assay, and O/TAN/39/2012, A/TAN/6/2013, SAT1/KEN/72/2010 and SAT2/TAN/19/2012 were used for the EA‐O, EA‐A, EA‐SAT 1 and EA‐SAT 2 assays, respectively, using a 10^–2^ dilution spiked into unpasteurized whole Jersey milk. Unpasteurized whole Jersey milk collected from a farm in the UK was used as a negative extraction control.

### 2.4 Statistical analysis

Associations were tested between the incidence of clinical FMD in the entire study area (either above or below the 2.5% household‐level FMD incidence threshold) and the rRT‐PCR testing, both at the study area level and within the individual milk pooling facility catchment area level. The spatial coordinates of surveyed farms were assigned to a facility catchment area using ArcGIS version 10.6.1 (Environmental Systems Research Institute, Inc.) based on approximate descriptions of the catchment areas from facility managers (Figure 1). Descriptive and statistical analyses were carried out using R 3.5.3 (R Core Team, 2019) within RStudio IDE (RStudio Team, 2019).

Mixed effect logistic regression analysis was performed including the milk pooling facility variable as a random effect on the intercept. This was implemented to examine associations between the binary outcome of the rRT‐PCR for weekly testing of the pooled milk samples in the entire study area (i.e. FMDV RNA detected: yes/no), and the following explanatory variables: (1) clinical FMD incidence, (2) tank volume, (3) number of farmers contributing to the facility, (4) the average number of adult female cows per farm, (5) the percentage of famers selling to a milk pooling facility and (6) the average milk yield per cow per day. Variables 2 and 3 utilized weekly data collected from the pooling facility at the time of milk sampling, using increments of 1,000 L for tank volume (variable 2) and 100 for the number of farmers contributing to the facility (variable 3). For FMD incidence (variable 1), farmers were asked whether they observed FMD on their farm, and when it occurred (in which half of the month) to create a binary variable. This was applied to each week of that half‐month, to enable comparison with the weekly rRT‐PCR of pooled milk (i.e. if FMD was identified on a farm in the second half of January, both weeks in this half‐month period were assigned as positive for clinical FMD). Variables 4, 5 and 6 utilized data from smallholder farmer surveys, so there were only three data points for weeks 3 (S1), 20 (S2) and 45 (S3). Therefore, for each variable, data points for the unrecorded weeks were predicted by linear interpolation.

A backward stepwise regression was performed to fit a final multivariate model, based on the results of a likelihood ratio tests to remove variables with a *p* value higher than 0.05.

## 3 RESULTS

### 3.1 Pooled milk

The average volume of milk recorded weekly in the tanks/cans over the entire study period was 3,019.2 (range [min–max]: 900.0–5,400.0), 1,469.8 (200.0–4,400.0), 237.5 (50.0–578.0), 473.1 (103.0–1,050.0) and 176.5 (90.0–270.0) L for milk pooling facilities A, B, C, D and E, respectively. Variabilities in milk supply were observed over the study period and are shown in Figure S1. This was likely influenced by the number of farmers contributing to the milk pools, which also varied with a similar pattern. Results for individual milk pooling facilities are shown in Figures S2 and S3. The average number of farmers contributing milk to A, B, C, D and E was 915 (range [min–max]: 450–1,500), 29 (17–50), 25 (10–60), 42 (11–57) and 22 (10–33), respectively. The average volume of milk sold to a pooling facility per farmer was 14.1 L (range [min–max]: 0.0–55.0) for the entire study area during the study period (Table 1).

**Table 1:** Descriptive summary of the study population

Milk pooling facility catchment area	*N* a	Mean number of cows per farm (range: min–max)	Mean number of female cows >2 years per farm (range: min–max)	Mean volume of milk (L) yield daily per farm (range: min–max)	Number of farms that sell to a milk pooling facility (%)^b^	Mean volume of milk (L) per farm sold to a milk pooling facility (range: min–max)^c^	Number of farmers reporting FMD cases (%)^c^	Number of farms vaccinated for FMD (%)^c^
Survey 1^d^								
A	45	4.4 (1.0–11.0)	2.7 (0.0–7.0)	8.5 (0.0–22.0)	13 (28.9)	6.8 (0.0–15.0)	N/A	N/A
B	40	7.9 (1.0–30.0)	3.9 (1.0–10.0)	11.6 (0.0–50.0)	19 (47.5)	13.4 (2.0–50.0)	N/A	N/A
C	74	4.9 (1.0–24.0)	2.2 (0.0–15.0)	9.7 (0.0–120.0)	10 (13.5)	7.9 (1.5–25.0)	N/A	N/A
D	35	6.3 (1.0–30.0)	3.4 (1.0–10.0)	13.4 (0.0–60.0)	9 (25.7)	18.9 (3.5–50.0)	N/A	N/A
E	8	3.5 (1.0–7.0)	1.9 (1.0–3.0)	6.6 (0.0–11.5)	0 (0.0)	0.0 (0.0–0.0)	N/A	N/A
Total study area	220	5.5 (1.0–42.0)	2.9 (0.0–15.0)	11.0 (0.0–140.0)	56 (25.5)	12.1 (0.0–55.0)	N/A	N/A
Survey 2								
A	39	5.5 (1.0–46.0)	2.8 (0.0–12.0)	6.8 (0.0–46.0)	7 (18.0)	11.0 (2.0–46.0)	2 (5.1)	19 (48.7)
B	16	10.6 (1.0–40.0)	3.7 (0.0–16.0)	7.4 (0.0–35.0)	2 (12.5)	10.0 (3.0–17.0)	0 (0.0)	5 (31.3)
C	35	6.5 (2.0–44.0)	2.9 (0.0–23.0)	8.6 (0.0–50.0)	1 (2.9)	50.0 (50.0 –50.0)	2 (5.7)	7 (20.0)
D	15	9.8 (2.0–40.0)	5.1 (0.0–16.0)	11.0 (0.5–35.0)	2 (13.3)	10.0 (3.0–17.0)	0 (0.0)	1 (6.7)
E	4	3.5 (2.0–5.0)	1.5 (1.0–2.0)	4.0 (2.0–6.0)	0 (0.0)	N/A	0 (0.0)	0 (0.0)
Total study area	117	7.3 (1.0–46.0)	3.4 (0.0–26.0)	8.9 (0.0–50.0)	14 (12.0)	14.8 (2.0–50.0)	4 (3.4)	42 (35.9)
Survey 3								
A	28	5.5 (1.0–17.0)	2.9 (0.0–10.0)	11.7 (0.0–45.0)	7 (25.0)	15.7 (5.0–40.0)	0 (0.0)	0 (0.0)
B	15	7.2 (1.0–30.0)	2.7 (1.0–5.0)	12.5 (0.0–77.0)	1 (6.7)	7 (7.0 –7.0)	0 (0.0)	4 (26.7)
C	41	6.6 (2.0–44.0)	3.1 (1.0–14.0)	16.0 (3.0–48.0)	2 (4.9)	23.5 (12.0–35.0)	0 (0.0)	0 (0.0)
D	17	4.2 (1.0–16.0)	2.3 (1.0–8.0)	15.2 (0.0–95.0)	2 (11.8)	6.5 (5.0–8.0)	0 (0.0)	2 (11.8)
E	8	6.9 (3.0–14.0)	3.1 (1.0–6.0)	20.3 (4.0–32.0)	0 (0.0)	N/A	0 (0.0)	0 (0.0)
Total study area	119	6.1 (1.0–44.0)	3.0 (0.0–16.0)	13.8 (0.0–95.0)	18 (15.1)	15.5 (4.0–40.0)	0 (0.0)	7 (5.9)

Note

N/A—not applicable as this occurred outside of the study period.

a Number of farms surveyed.

b Farms may sell to any milk pooling facility.

c S2 and S3 since the previous survey.

d S1 was a baseline survey, and results are described previously by Nyaguthii et al., 2019.

**Figure 2:**
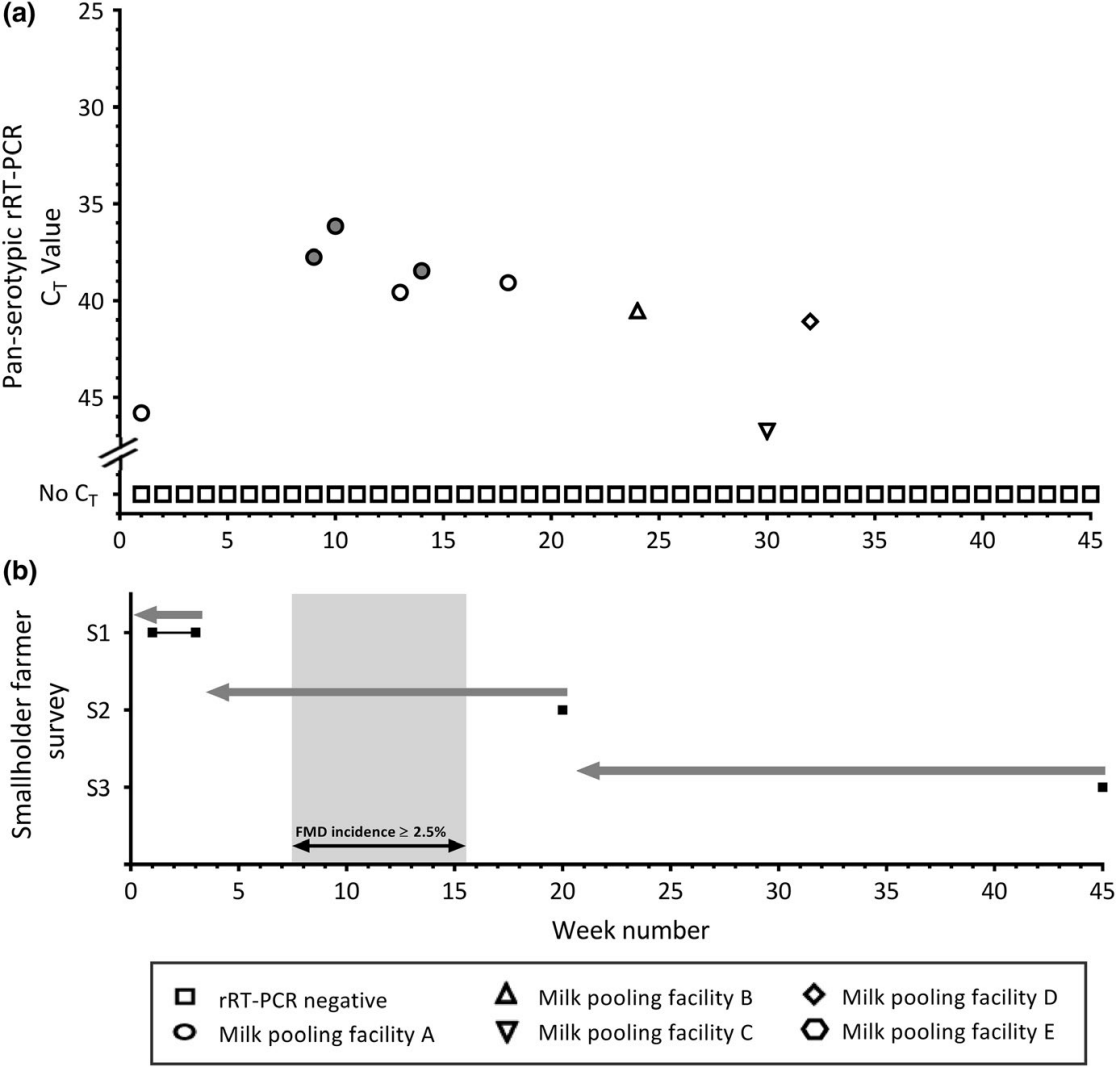
(a) Pan‐serotypic rRT‐PCR *C*_T_ values from pooled milk samples collected from milk pooling facilities A, B, C, D and E in Nakuru County, Kenya, over the 45‐week study period. Points with a grey centre represent samples that were also positive by the SAT 1 serotype‐specific assay (*C*_T_ value displayed is for the pan‐serotypic rRT‐PCR assay). (b) Black squares indicate time points of smallholder farmer surveys 1, 2 and 3. Grey arrows indicate the time period for which FMD incidence questions were based for each survey. Grey shading indicates time points where the FMD incidence was ≥2.5% (as reported by smallholder farmers during the household surveys). FMD, foot‐and‐mouth disease

A total of 219 pooled milk samples were collected from five facilities and tested using the pan‐serotypic rRT‐PCR. Milk samples were not collected on weeks 41–45 from facility B, due to a lack of milk supply, and on week 1 for facility E, as it was recruited a week later than the others. FMDV RNA genome was detected in 9/219 (4.11%) milk samples, six samples from facility A and one sample from each of facilities B, C and D (mean *C*_T_ value: 40.57, range [min–max]: 36.15–46.74) (Figure 2). Additionally, 3/9 samples (collected at facility A) with the strongest *C*_T_ values (<39) were also positive by the EA‐SAT 1 rRT‐PCR typing assay. No other serotypes were detected by the EA rRT‐PCR typing assays in the positive milk samples. The detection of SAT 1 in milk was concordant with results from clinical lesion material collected on the 27/01/2017 and submitted to the WRLFMD for confirmatory diagnostics, sequencing and phylogenetic analyses (WRLFMD, 2017a). However, milk samples that tested positive during the same period were from a different facility (A) than the catchment area where the clinical sample was collected from (D).

### 3.2 Cross‐sectional surveys

Descriptive data from each survey are detailed in Table 1 for the entire study area and also for the catchment areas of the individual milk pooling facilities. The number of smallholder farmers that took part in S1, S2 and S3 was 220, 117 and 119, respectively. Due to compilation of a combined catchment area and randomization of the spatial coordinates generated for surveying smallholder farms throughout the study area, some farms were located outside the boundaries of the individual catchment areas of any milk pooling facilities (Figure 1). Therefore, data from 47, 15, and 18 smallholder farms (for S1, S2 and S3, respectively) were not included in the descriptive data for individual catchment areas and only included in the descriptive data and regression analysis generalized to the entire study area (Table 1). Due to the overlap of some of the catchment areas, some farms were also included in the analysis for more than one individual catchment area.

The number of farmers in the entire study area that reported FMD on their farm during the 45 week study period was 4/456 (0.88%). All of the reported cases of FMD were in S2, when 4/117 (3.42%) farmers reported disease in their animals during either January or February (weeks 8–15), representing a household‐level incidence above 2.5% during those times (Figure 2).

The average volume of milk yield daily per farm was higher for S1 (11.0 L, 95% CI 9.0–13.1, *p* = .117) and S3 (13.8 L, 95% CI 11.1–16.5, *p* = .003) compared to S2 (8.9 L, 95% CI 7.0–10.7), consistent with the milk supply trends to facilities, and is likely related to a prolonged drought that took place during this time. For the entire study area, the largest percentage of farmers that sold to a facility at any time during the study period was 25.5% (S1), which was found to be lower during the subsequent surveys (S2 12.0%, *p* = .04; S3 15.1%, *p* = .20). The same pattern was observed for the individual catchment areas, where the largest number of farmers contributing at any time was in catchment area B (47.5%) recorded in S1. In the catchment area of facility E, none of the farms surveyed sold their milk to a pooling facility throughout the study period.

### 3.3 Correlation between FMDV RNA in milk samples from all pooling facilities and clinical FMD in the entire study area

Results from the rRT‐PCR were cross‐tabulated with the FMD clinical incidence in the entire study area (either above or below the 2.5% household‐level FMD incidence threshold), defined as the gold standard (Table 2). As each parameter was measured in different time periods (i.e. pooled milk was collected weekly, whereas the FMD incidence data collected in the surveys was recorded in half‐month increments), half‐month time periods were employed. Consequently, for the results of the rRT‐PCR, a binary variable was created, where a half‐month period was assigned as positive if there was a positive rRT‐PCR result in at least one week of that period. Therefore, by using the half‐month periods and taking the clinical incidence as reported by farmers in the whole study area as the gold standard, the pooled milk surveillance system had a sensitivity of 100% (95% CI 51.0%–100%) and specificity of 70.6% (95% CI 46.9%–86.7%) (Table 2). FMDV RNA was identified in four pooled milk samples collected during the period from January to February 2017 (weeks 8–15) when the clinical incidence at household level across the entire study area was significantly ≥2.5% (3.42%) (Figure 2). There were also instances where FMDV RNA was detected in the milk samples, but there were no corresponding reports by farmers (i.e. the household‐level incidence of FMD was not significantly above 2.5%).

**Table 2:** Comparison of the FMDV pan‐serotypic rRT‐PCR results of samples from all milk pooling facilities, with FMD incidence by farmer reports for the entire study area

	FMD incidence ≥ 2.5%		
	Yes	No	Total
Pan‐serotypic rRT‐PCR of pooled milk samples			
Positive^a^	4	5	9
Negative	0	12	12
Total	4	17	21

Note

Half‐month periods were assigned to the results of the rRT‐PCR of the pooled milk and deemed positive if there was a positive rRT‐PCR result in at least one week of that period. There were a total of 21 half‐month periods for the duration of the study.

Abbreviation: FMD, foot‐and‐mouth disease; FMDV, FMD virus.

a A positive pan‐serotypic rRT‐PCR result is defined as a *C*_T_ value of ≤50 in any week of the half‐month time period.

Both univariable analyses and the multivariable mixed effect logistic regression models were used to determine whether there was any association between the household‐level incidence being above 2.5% in the entire study area and the FMD rRT‐PCR results from pooled milk, further incorporating other predictors listed in Table 3. Based on the univariable analysis, there were a higher odds of observing a FMD‐positive rRT‐PCR result when the clinical incidence in the whole study area was ≥2.5% (OR = 4.21, 95% CI = 1.02–17.30, *p* = .046), when the volume of milk supplied to the facility increases (OR = 1.78 for each additional 1,000 L supplied, 95% CI = 1.25–2.54, *p* = .002) and when the number of farmers contributing to the facility increases (OR = 1.27 for each additional 100 farmers, 95% CI = 1.12–1.43, *p* = <.001). During multivariate model selection, after model simplification by removing non‐significant terms (*p* > .05), only the number of farmers that contribute to the facility (3) was retained as significant (OR = 1.27, 95% CI = 1.12–1.43, *p* = <.001), and there was no longer any association between the household‐level incidence being above 2.5% in the study area and the FMD rRT‐PCR result in the pooled milk.

**Table 3:** Univariate mixed effect logistic regression analysis for association with a positive FMD rRT‐PCR result for the total study area

Variable	Type of variable	Odds ratio (95% CI)	*p* Value
1. FMD incidence	Categorical	4.21 (1.02–17.31)	.046
2. Tank volume (per 1,000 L)	Continuous	1.78 (1.25–2.54)	.002
3. Number of farmers contributing to the facility (per 100 farmers)	Continuous	1.27 (1.12–1.43)	<.001
4. Average number of adult female cows per farm	Continuous	0.99 (0.26–3.78)	.990
5. Percentage of farmers selling to a milk pooling facility	Continuous	1.04 (0.96–1.11)	.358
6. Average milk yield per cow	Continuous	0.48 (0.15–1.49)	.203

Abbreviation: FMD, foot‐and‐mouth disease.

Univariable and multivariable logistic regression analysis was also carried out using the data for each individual catchment area only (data not shown). The only catchment area where any significant associations were observed was for facility A, where there was a higher odds of observing an FMD‐positive rRT‐PCR result when there was an increase in the number of farmers contributing to the tank (OR = 1.38 for each additional 100 farmers, 95% CI = 1.03–1.85, *p* = .031). Significant associations were not observed for any of the other catchment areas.

## 4 DISCUSSION

Previous studies have demonstrated the potential of using milk from individual animals as an alternative sample type for FMDV detection and surveillance (Armson et al., 2019) and that it is possible to detect FMDV in highly diluted milk samples from individual clinical cases (Armson et al., 2018). This pilot study aimed to expand on this work and explore the use of pooled milk as a cost‐effective, non‐invasive alternative sample matrix for the surveillance of FMD by comparing the household‐level incidence of clinical disease to FMDV RNA detection by rRT‐PCR from pooled milk facilities in an endemic region of Kenya.

According to data collected by milk pooling facilities, the number of farmers contributing milk fluctuated throughout the study period, with a similar pattern observed for the volume of milk supplied. There was a decrease in the output of milk supplied to all facilities in or after March 2017, likely due to the effects of a drought that occurred in the first quarter of 2017 (World Food Programme Kenya, 2017). This corresponded with data collected from the smallholder farmer surveys, demonstrating the lowest milk yield per farmer, and the lowest percentage of farmers supplying milk to a facility (12.0% for the entire study area) occurring at this time. The largest percentage of farmers that supplied milk to a facility at any time was 25.5% for the entire study area or 47.5% for an individual catchment area.

FMD virus RNA was detected in milk samples collected from pooling facilities with tanks containing up to 5,000 L. Additionally, typing assays confirmed the presence of SAT 1, which was concurrent with reports from clinical samples collected from reported outbreaks for confirmatory diagnostics (WRLFMD, 2017a). The average *C*_T_ values obtained for the positive milk samples were high (>36), likely due to the dilution factor of the samples, as some were collected from large pools (up to 5,000 L). This corresponds with previous limit of detection studies (Armson et al., 2018) that predicted similar *C*_T_ values (>30) for pools of this size. This study observed that an increase in tank volume was correlated with an increase in the number of farmers contributing milk, and consequently, it is probable that the likelihood of a FMD‐infected cow that supplied milk to one of these pools is increased, contrary to what might be expected based on rRT‐PCR test sensitivity. Univariable analysis supported this, suggesting a positive association between a FMD‐positive rRT‐PCR result in the pooled milk and the number of farmers contributing to the facility, and also with the volume of milk in the tank/cans at the time of sample collection. Based on these results, the likelihood of FMDV detection and therefore surveillance efficiency may be optimized by targeting sampling on large milk pooling facilities that have milk supplied from a large percentage of farmers in their catchment area.

During the study period, throughout the entire study area, there were four reports of FMD in the smallholder farmer surveys in facility catchment areas A and C, all during January and February 2017. As there was at least one FMD report in each half‐month period during these two months, the household‐level incidence in the whole of the study area was significantly ≥2.5%. This correlated with FMDV detected in a milk sample collected from at least one of the facilities in the study area in each of these half‐month periods; therefore, it could be assumed that the pooled milk surveillance system might be able to detect FMDV when the household‐level incidence is ≥2.5%. This was supported by univariable analysis which indicated a positive association between an FMD‐positive rRT‐PCR result when the clinical incidence in the entire study area was ≥2.5%. Although the sensitivity was 100% when using clinical reports from farmers as the gold standard, the authors acknowledge the limitations of using these half‐month time steps for this comparative analysis. These half‐month time periods for FMD reporting were used to simplify data recording which was based on farmer recall in the absence of written records.

Despite the high sensitivity, FMDV RNA was detected in five pooled milk samples that were collected when there were no clinical FMD reports, and the possibility that these results are due to laboratory contamination cannot be excluded. However, measures were implemented to minimize the likelihood of this occurring, and the laboratory methodology used in this study has been shown to be highly specific (data not shown). Additionally, as there were negative controls, and a high number of ‘negative’ samples where no amplification was observed on the rRT‐PCR assay, this further supports the theory that these positive milk samples are unlikely due to laboratory contamination or non‐specific amplification and that there may be further alternative explanations.

It is possible that the study was underpowered, due to limited resources available, and therefore, the clinical disease threshold of 2.5% was too high to robustly assess specificity. In future studies, a more precise evaluation of sensitivity and specificity of the pooled milk detection system may be achieved if surveys are powered to detect a lower threshold FMD incidence. Farmers in this region of Kenya had good knowledge of FMD (Nyaguthii et al., 2019), which was validated by the descriptions of clinical signs by farmers corresponding with the case definition. However, it is possible that mild clinical signs or sub‐clinical infection could reduce the likelihood of farmer reporting and provide explanation for instances where there were positive milk samples but no farmer reports of disease. Further investigation is required to determine the incidence of sub‐clinically infected animals in this region, for example by using serological surveys, and whether virus particles may be present in the milk of sub‐clinically infected animals (Sutmoller & Casas, 2002). Further investigation is also required into the impact of vaccination on FMDV excretion in milk. During the study period, vaccination was carried out in response to an outbreak. Whether vaccination in these herds may increase the likelihood of sub‐clinical infection is unknown, although there have been reports of sub‐clinical infection in vaccinated animals (Donaldson & Kitching, 1989; Hutber, Kitching, & Conway, 1999; Lyons et al., 2017) and virus excretion in the milk of apparently healthy vaccinated animals (Ahmed et al., 2017).

Of the milk samples positive by the pan‐serotypic rRT‐PCR assay, 3 were identified as SAT 1 but no amplification was observed in any of the EA typing rRT‐PCR assays for the other samples. Outbreaks due to the circulation of type O outside of the study area were reported in August 2017 (WRLFMD, 2017b). It is possible that these samples were at the limit of detection for the EA‐O rRT‐PCR typing assay, as this assay has been shown to have a slightly reduced analytical sensitivity compared with the pan‐serotypic rRT‐PCR assay (Bachanek‐Bankowska et al., 2016). It is also possible that another lineage of FMDV was also circulating in the region that cannot be detected by the EA typing assays used.

Several methodological issues arose during this study that may have affected the results of FMD clinical incidence and therefore the sensitivity and specificity estimations of the pooled milk surveillance system. The original aim of the study was to undertake smallholder farmer surveys within the catchment areas of the milk pooling facilities. Catchment areas were approximated by facility managers, and as some of the catchment areas either bordered or overlapped each other, a single spatial polygon was created to define the whole study area. It is unclear how precise these catchment areas were, as in some cases farmers from one catchment area reported supplying milk to a neighbouring catchment area. This may explain cases where there was positive report of FMD by a farmer in one catchment area, but there were no rRT‐PCR positive milk samples from the area's pooling facility in that time period (e.g. catchment area C). Additionally, some of the surveyed farms were located in more than one catchment area (due to overlap of the catchment areas), or none of the catchment areas (due to being between catchment areas), which may have led to bias in the descriptive data and analysis. Due to the absence of an available sampling frame, it was assumed that smallholder farmers were evenly distributed throughout the whole study area. This was a reasonable assumption based on the knowledge of the authors and animal health assistants in the area, although any disparity may have led to an inaccurate estimation of household‐level incidence. In addition, the intention of the study was to recruit milk pooling facilities that stored milk in bulk tanks for the collection of milk samples. However, three of the facilities either did not have, or were not using their bulk tanks, and instead pooled milk in 50‐L cans. The reasons for not using an existing bulk‐tank included a low milk supply and not being fully functional. Consequently, a small volume of milk from each can was pooled and mixed in order to obtain a sample representative of the whole milk pool from this facility. The authors recognize the limitations in this approach, and further sampling methodologies for facilities using cans should be explored.

This pilot study describes the rRT‐PCR testing of milk samples from milk pooling facilities as a simple surveillance approach for FMD in this endemic region of Kenya. Based on data from the entire study area, by utilizing the weekly collection of milk samples, it was possible to detect and type FMDV RNA by rRT‐PCR from milk pools of up to 5,000 L, when the FMD clinical incidence was ≥2.5%, and when fewer than 25% of farmers were selling their milk to these pooling facilities. Based on the results obtained in this study, further investigation is required to obtain a more precise correlation of household‐level incidence with pooled milk sample results, to fully assess the usefulness of this novel surveillance approach. With more resources available, this could be achieved by combining clinical surveys of FMD infection at the individual animal level and serological surveys with sufficient statistical powers to detect a low incidence of infection or disease. Additionally, the collection of pooled milk samples should be focussed on larger facilities, which have a large number of contributing farmers from the surrounding area. Furthermore, pooling systems higher up the dairy production chain should also be explored as a target for FMD surveillance, although the possible reduced ability in detecting FMDV RNA from milk samples after pasteurization. Follow‐on studies should also investigate the establishment of sentinel systems in the epidemiological surveillance of FMD, and how geographical limits that may encompass different farming practices may affect this solution.

In conclusion, this pilot study highlights that this novel, simple surveillance approach has the potential to address some of the well‐recognized limitations of more traditional surveillance methods in resource‐limited countries and to improve the capacity for surveillance which could contribute to informing and evaluating disease control policies in these endemic regions.

## ETHICAL APPROVAL AND CONSENT TO PARTICIPATE

Approval for this study was obtained from the Kenyatta National Hospital—University of Nairobi Ethics and Research Committee (reference: P301/04/2016).

## CONFLICT OF INTEREST

The authors declare no competing interests.

## Data Availability

The data that support the findings of this study are available in the Supporting Information of this article.
