# Supplementary material for: Utilizing milk from pooling facilities as a novel approach for foot‐and‐mouth disease surveillance
Source: Transbound Emerg Dis. 2020 Feb 4;67(4):1532–42. doi: 10.1111/tbed.13487 (PMC7384003; doi:10.1111/tbed.13487)
Supplement: Supplementary file 1 [file TBED-67-1532-s001.pdf]

Farm ID: ____________ **Date: ____/____/______**

Interviewer: ____________

THE Pirbright INSTITUTE

MINISTRY OF AGRICULTURE LIVESTOCK AND FISHERIES
DIRECTORATE OF VETERINARY SERVICES

Questionnaire: FQv1.2

Use of milk for foot-and-mouth disease surveillance: Field Validation in Endemic Settings

Follow-up questionnaire for interim surveys

1. Position at the farm: (please circle the correct choice)
   a. Owner
   b. Employee
   c. Milker
   d. Herdsman
   e. Manager
   f. Family member of main owner
   g. Other ____
2. GPS coordinates: longitude_____________ latitude_______________
3. How many years have the owners been in cattle farming? _______________years
4. How many cattle are currently on the farm? _______________
   Please complete the following grid to give the sex and age categories for cattle on the farm

Age of cow	Male	Female
< 6 months		
6-12 months		
1-2 years		
>2 years		

5. What other species are found in your farm (state number):
   a. Goats _________
   b. Sheep_________
   c. Pigs____________
   d. Donkeys_________

Farm ID: ____________ **Date: ____/____/______**

Interviewer: ____________

Risk factors

6. What grazing methods do you use for your animals?
    a. Zero grazing
    b. Within farm grazing
    c. Outside farm grazing
    d. Don't know
7. For within farm grazing, do animals graze close to the boundary of the farm?
    a. Yes (Always)
    b. Yes (Sometimes)
    c. Yes (Rarely)
    d. Never
    e. Don't know
8. For grazing outside the farm, do you use communal grazing fields to feed your animals?
    a. Yes (Always)
    b. Yes (Sometimes)
    c. Yes (Rarely)
    d. Never
    e. Don't know

Please complete table showing how grazing varies for each species:

**Species**	**Grazing method**<br>**1 = Zero; 2 = within farm grazing; 3 = outside farm grazing**	**Communal grazing?**<br>**Y=Yes; N=No**
Cattle		
Sheep		
Goat		
Pigs		

9. How is water provided for the animals?
    a. Stored rain water
    b. Piped water on farm
    c. Bore-hole on farm
    d. Private access to river
    e. Communal access to river
    f. Access to other communal access point (e.g. dam)
    g. Other (please state) ______________________

Farm ID: ____________ **Date: ____/____/______**

Interviewer: ____________

10. Which of the following communal grazing areas are used:
    a. Forests__________
    b. By roadsides________
    c. Fields post-harvest _______
    d. Other communal place ________
11. Do you employ any workers on the farm?
    a. Yes
    b. No
    c. Don't know
12. Do you share workers these worker(s) with other farms?
    a. Yes
    b. No
    c. Don't know
13. Do the worker(s) live on the farm?
    a. Yes
    b. No
    c. Don't know
14. Do the workers own any cattle/sheep/goats/pigs on other farms?
    a. Yes
    b. No
    c. Don't know
15. Do you share equipment with surrounding farms?
    a. Yes
    b. No
    c. Don't Know
16. How do you get replacement animals?
    a. Buying from markets
    b. Buying from other farms
    c. From my own animals
    d. Other (please state)
17. How do you breed your cattle?
    a. AI
    b. Own bull
    c. Shared bull
18. Do you use a dip or spray for tick control?
    a. Dip
    b. Spray
    c. Both
    d. Other (please state) _________________
    e. Don't know

19. How frequently do you apply tick control? ____________________________

Disease

20. Have you had any sick animals in the last four months?
    a. Yes
    b. No (Go to question 38)
    c. Don't know (Go to question 38)
21. What species have you seen illness in?
    a. Cattle (see question 21)
    b. Sheep (see question 26)
    c. Goats (see question 30)
    d. Pigs (see question 34)
    e. Other (please state) ___________________________________
22. What disease(s) have you seen in cattle? Ask as an open question. State how many months ago.
    a. FMD ___________________________months
    b. LSD ___________________________months
    c. CBPP ___________________________months
    d. ECF ___________________________months
    e. Bluetongue ___________________________months
    f. Anaplasmosis ___________________________months
    g. Mastitis ___________________________months
    h. Pneumonia ___________________________months
    i. Anthrax ___________________________months
    j. RVF ___________________________months
    k. Worms ___________________________months
    l. Not sure (go to Q22)
    m. Other (please state) ___________ ___________________________months
23. What signs did the animals show?
    a. Salivation
    b. Lameness
    c. Fever
    d. Inappetence
    e. Sudden death
    f. Diarrhoea/Gastrointestinal disease
    g. Respiratory disease
    h. Abortion
    i. Drop in milk yield
    j. Other (please state) ____

Farm ID: ____________ **Date: ____/____/______**

Interviewer: ____________

24. How many cattle were sick? _______
25. Of the animals affected how many were:

< 6 months	
6-12 months	
1-2 years	
>2 years	

26. What signs have you seen in **Sheep**? Ask as an open question. State how many months ago.
    a. PPR ___________________________months
    b. FMD ___________________________months
    c. Bluetongue ___________________________months
    d. Sheep pox ___________________________months
    e. Mastitis ___________________________months
    f. Respiratory disease ___________________________months
    g. Worms ___________________________months
    h. Abortion ___________________________months
    i. Sudden death ___________________________months
    j. Other (please state) _____________________ ___________________________months
27. What signs did the animals show?
    a. Salivation
    b. Lameness
    c. Fever
    d. Inappetence
    e. Sudden death
    f. Diarrhoea/Gastrointestinal disease
    g. Respiratory disease
    h. Abortion
    i. Skin lesions
    j. Other (please state) ____
28. How many sheep were sick? _______
29. Of the animals affected how many were:

	**Sick**
< 12 months	
1-2 years	
>2 years	

Farm ID: ____________ **Date: ____/____/______**

Interviewer: ____________

30. What signs have you seen in **Goats**? Ask as an open question. State how many months ago.
    a. PPR ____________________________months
    b. FMD ____________________________months
    c. CCPP ____________________________months
    d. Goatpox ____________________________months
    e. Bluetongue ____________________________months
    f. Mastitis ____________________________months
    g. Respiratory disease ____________________________months
    h. Worms ____________________________months
    i. Abortion ____________________________months
    j. Sudden death ____________________________months
    k. Other (please state) ____
31. What signs did the animals show?
    a. Salivation
    b. Lameness
    c. Fever
    d. Inappetence
    e. Sudden death
    f. Diarrhoea/Gastrointestinal disease
    g. Respiratory disease
    h. Abortion
    i. Other (please state) ____
32. How many goats were sick? _______
33. Of the animals affected how many were:

	**Sick**
< 12 months	
1-2 years	
>2 years	

34. What signs have you seen in **pigs**? Ask as an open question. State how many months ago.
    a. FMD ____________________________months
    b. Respiratory disease ____________________________months
    c. Worms ____________________________months
    d. Abortion ____________________________months
    e. Sudden death ____________________________months
    f. Other (please state) ______________________ ____________________________months

Farm ID: ____________ **Date: ____/____/______**

Interviewer: ____________

35. What signs did the animals show?
    a. Salivation
    b. Lameness
    c. Fever
    d. Inappetence
    e. Sudden death
    f. Diarrhoea/Gastrointestinal disease
    g. Respiratory disease
    h. Abortion
    i. Other (please state) ____
36. How many pigs were sick? _______
37. Of the animals affected how many were:

	**Sick**
< 12 months	
1-2 years	
>2 years	

38. Did you call a vet or animal health assistant when you animals were sick?
    a. Yes (state disease event)
    b. No
    c. Don't know
39. Was this a private or government employee?
    a. Government
    b. Private
    c. Don't know

Farm ID: ____________ **Date: ____/____/______**

Interviewer: ____________

Local disease reports and vaccination

40. Have you heard reports of any other diseases in the local area in the last four months?
    a. Yes
    b. No (Go to 42)
    c. Don't know (Go to 42)
41. Which diseases did you hear about (state how many months ago)?
    a. FMD ____________________________months
    b. LSD ____________________________months
    c. PPR ____________________________months
    d. ECF ____________________________months
    e. Anaplasmosis ____________________________months
    f. Mastitis____________________________months
    g. Pneumonia ____________________________months
    h. Anthrax ____________________________months
    i. Bluetongue ____________________________months
    j. Other (please state) ________________________________
    k. Don't know
42. Has there been any vaccination on the farm in the last four months?
    a. Yes
    b. No
    c. Don't know
43. Which disease(s) have been vaccinated for (state how many months ago)?
    a. Foot-and-mouth disease __________________________months
    b. Lumpy Skin disease __________________________months
    c. PPR __________________________months
    d. Anthrax __________________________months
    e. ECF _______________ __________________________months
    f. Other (please state) _______________ __________________________months
    g. Don't know
44. Who did the vaccination?
    a. Private animal health assistant/veterinarian
    b. Government animal health assistant/veterinarian
    c. Government
    d. Other (please state) ____

Farm ID: ____________ **Date: ____/____/______**

Interviewer: ____________

Milk Production and Delivery

45. How much milk did your farm produce yesterday? _________ litres
46. How much of this did you sell? _________ litres
47. How many cows were lactating? _________cows
48. To whom do you sell your milk?
    a. Neighbours
    b. Local hotels
    c. Hawkers
    d. Direct to co-operative or other pooling facility
    e. Other (please state) ________________________________
49. What is the name of the co-operative/pooling facility? __________________________
50. What does the hawker do with the milk?
    a. Sells to private places
    b. Sells to milk pooling facility
    c. Other (please state) _________________________
    d. Don't know
51. If the farmer had FMD in the last 4 months, what did they do with the milk while the animals were sick? Sell it to the cooperative society
    a. Consume it at home
    b. Boil and consume at home
    c. Sell it to others
    d. Dispose of it
    e. Give to other animals (e.g. dogs)
    f. Give it to calves
    g. Give it to calves (after boiling)
    h. Other (please specify) _____________________________
